# Impact of the SARS-CoV-2 pandemic and first lockdown on pregnancy monitoring in France: the COVIMATER cross-sectional study

**DOI:** 10.1186/s12884-021-04256-9

**Published:** 2021-11-30

**Authors:** Alexandra Doncarli, Lucia Araujo-Chaveron, Catherine Crenn-Hebert, Virginie Demiguel, Julie Boudet-Berquier, Yaya Barry, Maria-Eugênia Gomes Do Espirito Santo, Andrea Guajardo-Villar, Claudie Menguy, Anouk Tabaï, Karine Wyndels, Alexandra Benachi, Nolwenn Regnault

**Affiliations:** 1 Santé publique France, French national public health agency, Non-Communicable Diseases and Trauma Division, Perinatology, Early childhood and Mental Health Unit, 14, rue du Val d’Osne, F-94415 Saint-Maurice, France; 2 Department of Gynecology and Obstetrics, Louis Mourier University Hospital, AP-HP, Colombes, France; 3 Santé publique France, French national public health agency, Data processing, support and analysis department, Saint-Maurice, France; 4 Santé publique France, French national public health agency, Alert and crisis department, Saint-Maurice, France; 5 Santé Publique France, French national public health agency, Hauts-de-France regional office, Saint-Maurice, France; 6 Division of Obstetrics and Gynecology, Antoine Béclère Hospital, AP-HP, Clamart, France; 7 Paris Saclay University, Clamart, France

**Keywords:** Pregnant women, SARS-CoV-2, Lockdown, Pregnancy monitoring

## Abstract

**Background:**

In the context of the severe acute respiratory syndrome coronavirus 2 (SARS-CoV-2) pandemic, consultations and pregnancy monitoring examinations had to be reorganised urgently. In addition, women themselves may have postponed or cancelled their medical monitoring for organisational reasons, for fear of contracting the disease caused by SARS-CoV-2 (COVID-19) or for other reasons of their own. Delayed care can have deleterious consequences for both the mother and the child. Our objective was therefore to study the impact of the SARS-CoV-2 pandemic and the first lockdown in France on voluntary changes by pregnant women in the medical monitoring of their pregnancy and the associated factors.

**Methods:**

A cross-sectional study was conducted in July 2020 using a web-questionnaire completed by 500 adult (> 18 years old) pregnant women during the first French lockdown (March–May 2020). A robust variance Poisson regression model was used to estimate adjusted prevalence ratios (aPRs).

**Results:**

Almost one women of five (23.4%) reported having voluntarily postponed or foregone at least one consultation or pregnancy check-up during the lockdown. Women who were professionally inactive (aPR = 1.98, CI95%[1.24–3.16]), who had experienced serious disputes or violence during the lockdown (1.47, [1.00–2.16]), who felt they received little or no support (1.71, [1.07–2.71]), and those who changed health professionals during the lockdown (1.57, [1.04–2.36]) were all more likely to have voluntarily changed their pregnancy monitoring. Higher level of worry about the pandemic was associated with a lower probability of voluntarily changing pregnancy monitoring (0.66, [0.46–0.96]).

**Conclusions:**

Our results can guide prevention and support policies for pregnant women in the current and future pandemics.

**Supplementary Information:**

The online version contains supplementary material available at 10.1186/s12884-021-04256-9.

## Background

Data from previous coronavirus outbreaks in 2002 and 2013 showed that pregnancy was a risk factor for severe forms of associated respiratory diseases. More specifically, SARS-CoV-1 and Middle East respiratory syndrome-related coronavirus were associated with significant acute respiratory distress syndrome [1, 2]. This fact, together with recommendations of learned societies [3, 4], prompted several countries, including France, to declare in March/April 2020 that pregnant women should be considered a population at greater risk of severe forms of COVID-19, the disease caused by SARS-CoV-2 [5–9]. In the absence of vaccines and effective pharmaceutical treatments at that time, most governments decided to reduce the spread of the virus by implementing strict lockdowns of their entire population for several months. These actions together with to the increased influx of patients suffering from COVID-19 brought about major changes in the organisation of health systems [10–12], including the organisation of hospital gynaecological departments [10–13]. In the United States (U.S.) a longitudinal study reported a decrease of 40% in on-site abortion during the first trimester of pregnancy between February and June 2020. In addition, a decrease in on-site consultations for abortion follow-up was observed, prompting an increase in teleconsultations and medical abortion at home [13]. Another U.S. study, conducted between mid-March and mid-May 2020 showed that nearly one-third of pregnancy monitoring visits were modified, cancelled or rescheduled [14]. In France, a longitudinal study on the surgical management of gynaecological cancers reported a change in medical management for 27% of its participants, including 23.2% for whom surgery was either postponed or cancelled due to the influx of patients infected with SARS-CoV-2 during the first lockdown [15], which took place between 16 March and 11 May 2020.

With regard to pregnancy, monitoring consultations were initially deprogrammed by French health professionals before official guidelines recommended that follow-up be maintained and reorganised [3, 12, 16, 17]. The French healthcare system adapted very quickly to the crisis, offering 100% reimbursed tailored teleconsultation (video and telephone-based consultations) to pregnant women [18]. Maternity wards and private offices also changed pregnancy monitoring practices. More specifically, partners were not allowed to be present during consultations, obstetric examinations or during hospitalization for childbirth, except under certain conditions [19]. Only partners were allowed to visit after childbirth [19].

In addition to the reorganisation of the health sector, some women voluntarily (i.e., spontaneously) modified their medical monitoring for different reasons, for example organisational scheduling [20].

Any postponement or foregoing of consultations or examinations in the context of pregnancy is of particular concern for the health of both the mother and child, as screening must be performed within specific time windows [21]. More specifically, failure to monitor foetal weight gain, screen for gestational diabetes and hypertension, has deleterious consequences for the newborn [22–24].

In the context of the SARS-CoV-2 pandemic, it is essential that the health management of pregnant women be continually adapted to best meet the changing needs in this dynamic context. To do this, it is necessary to understand the reasons why pregnant women voluntarily change their pregnancy monitoring.

Our objective was to study i) the effects of the SARS-CoV-2 pandemic and France’s first associated lockdown on the frequency of voluntary changes in pregnancy monitoring by women during the country’s first lockdown, and ii) associated factors.

## Methods

### Study population (Covimater)

Our sample comprised 500 adult women who were pregnant during the first lockdown in France (17 March - 11 May 2020). Participants were 18 years old and over and residents in metropolitan France. We excluded two groups of women who were pregnant during the lockdown but with limited exposure to it: those who delivered in the two first weeks of the lockdown and those whose first week of gestation began during the last two weeks of the lockdown (deducted from the expected date of delivery reported by the women).

### Survey methodology

At our request, a service provider (BVA group) interviewed its unpaid pre-pandemic panel of 15,000 future parents or parents of children under 3 years of age in order to create a pseudonymised non-probability sample of 500 adult pregnant women who met the inclusion criteria and volunteered to participate in our survey. Covimater used quotas sampling, whereby the study sample is assigned a structure similar to that of the target population (i.e., all pregnant women) in order to tend towards representativeness. The population of parents of children under one year old - as per the National Institute of Statistics and Economic Studies 2016 census - was used to set the quotas [25]. Indeed, the latter was a good proxy for our target population of pregnant women in France. Only the quotas for mothers were used to calculate weightings using Newton’s algorithm [26]. Specifically, these quotas comprised age group, socio-professional category (SPC), region of residence, size of urban area, and parity. Eligible women were invited to answer a web-based questionnaire between 6 and 20 July 2020, which collected socio-demographic/economic data, pandemic and lockdown-related data, participants’ perceptions of the pandemic, data on their pregnancy and health, and on pregnancy monitoring during the first lockdown (see Additional file 1). We compared our sample to another data source (the National Medical and Administrative Database) in order to validate its representativeness. No significant difference in available data for age group, region of residence or parity was observed between women participating in Covimater and the whole population of women in France who gave birth in a hospital maternity ward (i.e., 99% of French pregnant women population [27]). Our study shows, with a power of 99%, a difference of at least 20% concerning the variable of interest (see definition below) between two subgroups of balanced/unbalanced women.

### Changes in pregnancy monitoring at the initiative of pregnant women

For the present study, women who voluntarily changed their pregnancy monitoring during the lockdown were defined as those who reported at least one of the following in the questionnaire: (i) foregoing pregnancy examinations, (ii) voluntarily postponing or cancelling pregnancy consultations, (iii) delaying the start of monitoring (i.e., not starting monitoring despite a gestational age of over 15 weeks) [27].

The reasons for changes in pregnancy monitoring were explored through 13 binary questionnaire items covering different themes (lockdown, SARS-CoV-2 infection, and organisational problems, specifically personal and healthcare-based).

### Covariates

Explanatory variables were divided into five main themes:

#### Demographic and socio-economic

Age, socio-professional category (SPC) reduced into SPC+ (self-employed women, managers, intermediate professions), SPC- (employees, blue-collar workers) and Inactive (students and other professionally inactives), education level (equal to or higher than secondary school diploma, lower than secondary school diploma), perceived financial situation (comfortable, just getting by, difficult to make ends meet).

#### Pandemic and lockdown-related

Child(ren) under six years of age (i.e., younger than required school age in France) in the household during the lockdown, SARS-CoV-2 healthcare system severity as reported by the Ministry of Health on 1 May 2020 in their region of residence (coded as green, orange or red, reflecting increased epidemic pressure on the healthcare system) [28], professional workload (did not work, lighter/same than usual, heavier than usual), access to a private/common outdoor space, self-perceived social support (i.e., from family, friends, etc.) (Very good, Good, Little or none), having experienced serious arguments and/or a climate of violence (Very-often/Often, Sometimes/Rarely, Never), having had COVID-19 type symptoms, family member or friends diagnosed with COVID-19 or had symptoms suggestive of the disease.

#### Perception of the epidemic

Participants’ perceived general worry about the pandemic situation in France (scale from 0, not at all worried to 10, very worried). A dichotomous variable was then created with 7/10 as the thresholds corresponding to the average worry observed (7.0 +/− 0.1).

#### Pregnancy and health

Parity, gestational age at the end of lockdown, childbirth (during or after first lockdown), at least one pre-existing chronic disease or pregnancy-related pathology, overweight/obesity status before pregnancy (Body Mass Index ≥25 kg/m^2^).

#### Pregnancy monitoring during first lockdown

Unsuccessful attempts to have an exchange with healthcare professionals about the course of pregnancy/childbirth during pandemic, change in health professional from the one who usually followed them, teleconsultation (video or telephone) for pregnancy monitoring, absence of partner/person providing support from at least one pregnancy consultation/examination due to pandemic related restrictions, childbirth preparation sessions (video or telephone).

### Statistical analysis

A robust variance Poisson regression model was used to estimate unadjusted and adjusted prevalence ratios (aPR) [29] for voluntary changes in pregnancy monitoring. Factors associated with this outcome which had a *p*-value< 0.20 in bivariate analysis or which were judged to be clinically relevant based on the literature (gestational age at the time the study questionnaire was completed, gestational age at the end of the lockdown period, parity) were introduced into the multivariate model. When several variables were possibly collinear, the model with the best likelihood score (lowest Bayesian Information Criterion) was selected. Fractional polynomials showed a linear relationship between continuous variables included in the models and the studied prevalence of the outcome. The final model included the variables independently associated with the variable of interest (*p*-value< 0.05) after epidemiological reflection and according to the clinical relevance of each variable at each step of the procedure. A manual stepwise descending approach was applied.

Estimates of aPR, their 95% confidence intervals (95% CI) and associated *p*-values were presented. As indicated by Zou, PRs are interpreted in the same way as relative risks [30].

All statistical analyses were performed using Stata software® version 14.2 (Stata Corp., College Station, TX, USA).

## Results

### Characteristics of women included in Covimater (Table 1)

The mean age was of the Covimater study sample (*n* = 500) was 31.4 years (sd = 5.1). Four-fifths (78.1%) had a secondary school diploma or higher level of education, 36.1% were classified SPC-, 25.5% were Inactive, 31.7% declared they just got by financially, while 19.1% reported that they could not make ends meet.

**Table 1:** Description of pregnant women who participated in the Covimater survey (*n* = 500), France (July 2020)

	N (%)*		95%CI**
**Demographic and socio-economic characteristics**			
Age (in years)			
18–24	53	(10.7)	7.4–15.2
25–34	323	(64.6)	59.7–69.2
35–49	124	(24.7)	21.1–28.8
Socio-professional category (SPC)^a^			
SPC +	192	(38.4)	33.9–43.2
SPC -	180	(36.1)	31.8–40.6
Inactive	128	(25.5)	20.5–31.2
Educational level			
Equal to or higher than secondary school diploma	391	(78.1)	73.6–82.1
Lower than secondary school diploma	109	(21.9)	17.9–26.4
Perceived financial situation			
Comfortable	246	(49.2)	44.2–54.2
Just getting by	159	(31.7)	27.2–36.6
Difficult to make ends meet	95	(19.1)	15.2–23.7
**Pandemic and lockdown related variables**			
Child(ren) under 6 years of age in the household during the lockdown	234	(46.8)	41.8–51.8
SARS-CoV-2 healthcare system severity (colour-coded) for the region of residence^b^			
Green zone	127	(25.4)	21.1–30.2
Orange zone	150	(30.0)	25.7–34.7
Red zone	223	(44.6)	39.7–49.6
Professional workload			
Did not work	351	(70.1)	65.7–74.2
Lighter or same as usual	85	(17.1)	14.0–20.7
Heavier than usual	64	(12.8)	10.1–16.0
Self-perceived social support			
Very good	180	(36.0)	31.3–40.9
Good	231	(46.1)	41.2–51.1
Little or none	89	(17.9)	14.5–21.8
Serious disputes or violence			
Very-often / Often	11	(2.3)	1.1–4.6
Sometimes / Rarely	129	(25.8)	21.7–30.4
Never	360	(71.9)	67.2–76.2
Having had COVID-19 type symptoms	92	(18.4)	14.9–22.6
Family member or friends diagnosed with COVID-19 or had symptoms suggestive of the disease	171	(34.2)	29.7–39.0
**Perception of the pandemic**			
General worry score for the SARS-CoV-2 pandemic (max.10; *n* = 485; No documented data = 15) > 7/10	234	(48.3)	43.3–53.3
**Pregnancy and health**			
Primiparous	203	(40.6)	35.8–45.6
Gestational age (weeks) at the end of first lockdown^c^			
< 10	34	(6.8)	4.7–9.8
10–20	177	(35.4)	30.8–40.3
20–30	180	(36.1)	31.4–41.0
30–40	77	(15.4)	12.1–19.4
> 40	32	(6.3)	4.3–9.2
Childbirth			
During lockdown	34	(6.8)	4.7–9.8
After lockdown	466	(93.2)	90.2–95.2
Pre-existing chronic disease(s)^d^	152	(30.3)	25.8–35.1
Overweight / Obesity^e^	212	(42.4)	37.5–47.4
Pregnancy-related pathology(ies)^f^	119	(23.7)	19.9–28.0
**Pregnancy monitoring during first lockdown**			
Having an unsuccessful attempts to exchange with health professionals about course of pregnancy/childbirth during pandemic	205	(41.0)	36.1–46.1
Change of health professional than the referring professional^g^	74	(14.9)	11.7–18.8
Teleconsultations (video or telephone) for pregnancy monitoring	197	(39.4)	34.6–44.4
Absence of partner/person providing support from a consultation/examination	459	(91.8)	88.8–94.1
Childbirth preparation sessions (video or telephone)	76	(15.2)	12.0–19.1
Modification of pregnancy monitoring at the initiative of a health professional^h^	182	(36.3)	31.6–41.3
Modification of pregnancy monitoring at the initiative of the women^i^	117	(23.4)	18.8–27.7

* Weighted and rounded values using Newton’s algorithm [26]

** 95% Confidence Interval

^a^ Women on maternity leave and unemployed women were classified according to their current SPC category or their most recent category prior to ending work, respectively.

^b^ Estimated by the Ministry of Health on 1 May 2020 on the basis of two variables: i) Virus circulation level (i.e., percentage of emergency room admissions for suspected COVID-19) and ii) Strain on hospital intensive care unit capacity (i.e.,occupancy rate of intensive care beds by patients with COVID-19), coded as green, orange or red, reflecting increased epidemic pressure on the healthcare system [28]

^c^ At the end of the first lockdown (11 May 2020) or at the date of childbirth if women gave birth during lockdown

^d^ Diabetes, Overweight/Obesity status before pregnancy, High Blood Pressure, Asthma, Cardiac condition, Autoimmune disease, mental illness, etc.

^e^ Body Mass Index≥25 kg/m2

^f^ Gestational diabetes, pre-eclampsia, preterm labour, gestational hypertension, etc.

^g^ performed in a sub-group of pregnant women having start monitoring

^h^ Modification was to postpone/cancel pregnancy monitoring

^i^ Modification was to postpone/forego /not start monitoring despite a gestational age of 15 weeks (see definition of the variable of interest in Methods section)

From a medical perspective, 42.4% had overweight or obesity before pregnancy, and 23.7% had pregnancy-related pathologies, notably gestational diabetes (12.6%), preterm labour (5.9%), and gestational hypertension (1.6%) (data not shown). Finally, 17.9% perceived receiving little or no social support during the lockdown, and nearly 28% experienced serious arguments and/or a climate of violence during the same period.

### Pregnancy monitoring during the first lockdown (Table 1)

A total of 14.9% of women reported that they had been followed by a professional other than the one who usually followed them, 39.4% reported that they had teleconsultations, and 91.8% reported that their partner or a person providing support had not been allowed to attend at least one pregnancy check-up or consultation due to pandemic-related restrictions. In addition, 41% indicated that they unsuccessfully sought exchanges about the SARS-CoV-2 pandemic and their pregnancy with healthcare professionals.

Of the pregnant women who indicated that they had not started their pregnancy monitoring during the lockdown, the majority (63.7%) had a gestational age of over 15 weeks, indicating a delay in the management of their pregnancy.

### Voluntary changes in pregnancy monitoring

Just under half of the study sample (48.9%) reported having at least one consultation or pregnancy check-up postponed or cancelled during the lockdown, whether on their own initiative (23.4%) and/or the initiative of the hospital and/or a health professional (36.3%).

**Fig. 1:**
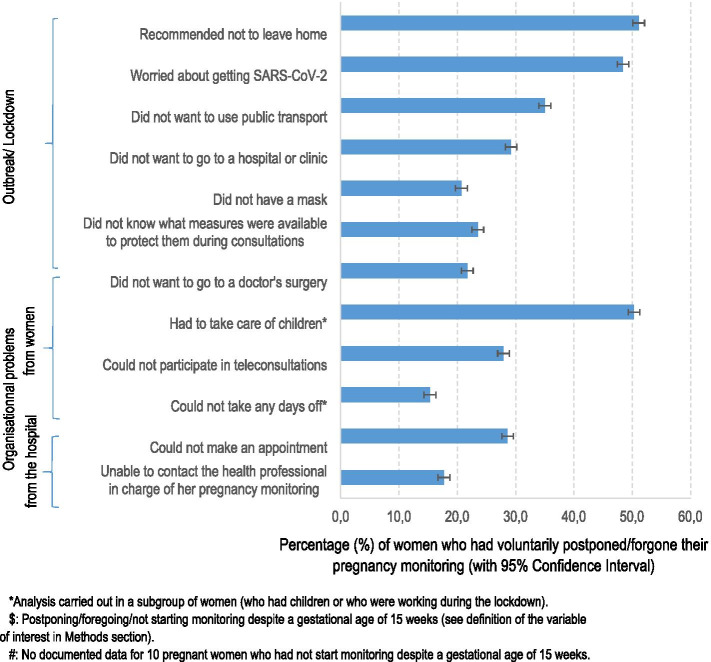
Reasons given by pregnant women^#^ to explain a voluntarily change^$^ in pregnancy monitoring during lockdown

The most frequent reasons participants gave for voluntary changes to their pregnancy monitoring were related to the pandemic and lockdown (i.e., fear of being infected with SARS-CoV-2; compliance with restrictions on movement), to personal organisational problems (e.g., caring for other children), and hospital problems (e.g., inability to make an appointment with a health professional) (Fig. 1).

**Fig. 2:**
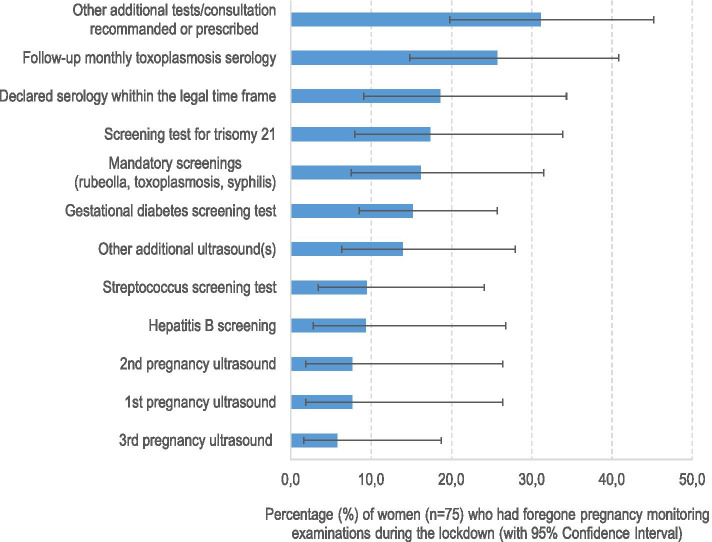
Foregoing of pregnancy monitoring examinations during the lockdown, Covimater survey; (*n* = 75), metropolitan France, 2020

Among women who declared foregoing at least one pregnancy monitoring examination (*n* = 75) as a direct result of the lockdown, almost one-third forewent i) supplementary prescribed or recommended pregnancy monitoring examinations/consultations (31.1%, CI95%[19.8–45.2]) or ii) Trisomy 21 screening (29.2% [17.1–45.1] regardless of gestational age (data not shown); 17.4% [7.9–33.9] before 16 gestational weeks), iii) one in four reported not having monthly toxoplasmosis serology (25.7%, [14.8–41] (Fig. 2).

After adjusting for age, gestational age and parity during the first lockdown, being inactive (RPa = 1.98, CI95%[1.24–3.16]), having experienced violence (1.47, [1.00–2.16]), having felt little or no support (1.70, [1.07–2.71]), and having changed healthcare professional (1.57, [1.04–2.36]) were all independently and significantly associated with a voluntary change in pregnancy monitoring. Conversely, higher level of worry about the pandemic was inversely associated with voluntary change in pregnancy monitoring (0.66 [0.46–0.96]) (Table 2).

**Table 2:** Factors associated with the prevalence of voluntary changes^a^ in women’s pregnancy monitoring in France, (07/2020)

	Obs. (n %)^*^		Voluntary changes in medical monitoring of pregnancy initiated by pregnant women (***n*** = 117)^a^			
			Yes (n %)^*^		Adjusted PR [95% CI] ^**^	***p***-value^**^
Age (in years)	31.4	(5.1)	32	(5.6)	0.99 [0.96–1.03]	0.77
Socio-professional categories^b^						
SPC+	192	(38.4)	38	(19.8)	1	
SPC-	180	(36.1)	29	(16.1)	0.71 [0.46–1.12]	0.14
Inactive	128	(25.5)	50	(39.1)	**1.98 [1.24–3.16]**	**0.004**
Pregnancy term^c^	23.5	(9.0)	24	(8.9)	1.00 [0.98–1.02]	0.84
Parity						
Primiparous	203	(40.6)	48	(23.6)	1	
Multiparous	297	(59.4)	69	(23.2)	0.96 [0.64–1.43]	0.84
Level of perceived social support received during the lockdown						
Very good	180	(36)	40	(22.2)	1	
Good	231	(46.1)	48	(20.8)	0.95 [0.62–1.46]	0.82
Little or none	89	(17.9)	29	(32.6)	**1.70 [1.07–2.71]**	**0.03**
Serious disputes or violence during the lockdown						
No	360	(71.9)	74	(20.6)	1	
Yes	140	(28.1)	43	(30.7)	**1.47 [1.00–2.16]**	**0.05**
Perceived general worry about the SARS-CoV-2 pandemic in France (max.10)^d^						
Score less than or equal to 7	251	(51.7)	67	(26.7)	**1**	
Score above 7	234	(48.3)	42	(17.9)	**0.66 [0.46–0.96]**	**0.03**
Change in health professional for pregnancy monitoring during the lockdown						
No	426	(85.1)	90	(21.1)	1	
Yes	74	(14.9)	27	(36.5)	**1.57 [1.04–2.36]**	**0.03**

* Weighted and rounded values using Newton’s algorithm [26] for discrete or qualitative variables. For continuous variables (age, pregnancy term), mean (standard deviation) were presented

** Adjusted Prevalence Ratio (aPR), Confidence Interval 95% (95%CI) and *p-*value obtained with robust variance Poisson regression model

^a^ Postponing/foregoing/not starting monitoring despite a gestational age of 15 weeks (see definition of the variable of interest in Methods section)

^b^ Women on maternity leave and unemployed women were classified according to their current SPC category or their most recent category prior to ending work respectively

^c^ At the end of the first lockdown (11/05/2020)

^d^ 15 women did not document their worry score

## Discussion

Just under half the study sample (48.9%) reported at least one consultation or pregnancy check-up being postponed or cancelled during the first lockdown, whether on their own initiative (23.4%) and/or the initiative of the hospital and/or that of a health professional (36.3%).

Women who i) were inactive, ii) perceived received little or no social support, iii) experienced violence, and whose iv) healthcare professional changed during the first lockdown, were all significantly more likely to voluntarily change their pregnancy monitoring. Conversely, worry about the pandemic was inversely associated with changing pregnancy monitoring.

Covimater’s results show an association between violence and a voluntary change in pregnancy monitoring. The percentage of pregnant women who reported experiencing violence or serious arguments during the first lockdown was high (28.1%), but significantly lower than that obtained for women of childbearing age (18–49 years) in CoviPrev, a French general population-based repeated cross-sectional study which used the same methodology as Covimater and conducted data-collection waves at the same time (CoviPrev study, 28.1% vs. 32.9%, *p* = 0.03) [31]. This result is in line with several studies suggesting that the prevalence of violence on women during pregnancy is no higher than in other situations. However, there is no international consensus about whether the risk of violence is higher in pregnant women than in women who are not pregnant [32–34]. Violence during pregnancy not only negatively impacts mothers’ health, but also that of their unborn children. It also impacts success of antenatal care. Furthermore, violence is significantly associated with an increased risk of obstetrical complications [35–37]. In terms of antenatal care, a survey held by the World Health Organisation in Tanzania studying domestic violence on women showed that it was significantly associated with fewer consultations for antenatal care because partners prevented or discouraged women from having them [34]. Efforts to detect violence against pregnant women at an early stage must be continued in order to prevent its harmful impact on health.

In Covimater, perceiving little or no support during the lockdown was associated with voluntary change in pregnancy monitoring. These results reflect findings from the 2010 French National Perinatal Survey (NPS), where women who declared having no social support were significantly more likely to forego care [38]. The perception of receiving little support may have been accentuated by the fact that during the first lockdown, in many maternity hospitals and private practices in France, neither partners nor people providing support to pregnant women were allowed to be present at consultations, obstetrical examinations, and hospitalisation for childbirth, except under certain conditions [19]. Only partners were allowed to visit after childbirth.

In our analyses, a change in health professional during the lockdown was associated with a higher likelihood of voluntarily postponing or foregoing pregnancy monitoring. As reported in several studies showing the importance of the patient/caregiver relationship in medical follow-up (in terms of treatment adherence, health examinations, etc.), it seems fundamental to ensure that the monitoring of pregnant women is as personalized as possible in the context of an ongoing pandemic.

In our study, women who had a higher worry score about the pandemic were less likely to change their pregnancy monitoring. This result suggests the need to communicate with pregnant women with a double objective: i) to avoid any increase in existing worry about the pandemic, and ii) to foster their adherence to health authorities’ recommendations concerning uninterrupted pregnancy monitoring. To ensure the quality and regular updating of information received by pregnant women, it is important to involve health care providers so that they can inform or direct their patients to reliable and responsive sources of information [39]. French laws for patients’ rights and the Public Health Code stipulate that patients have the right to have access to information [40] and that doctors must inform them of advances in science according to their needs. Access to reliable information is therefore an essential element in effective patient follow-up.

Finally, in line with Ancelot et al.’s findings in the NPS study in France in 2010 [38], having a chronic illnesses or a pregnancy-related illness was not significantly associated with a voluntary change in pregnancy monitoring in Covimater. Furthermore, participants in Covimater with a deteriorated psychological state during the first lockdown were not more likely to change their pregnancy monitoring than those with no such condition (*p*-value = 0.89).

In addition to characterising women with a higher prevalence of modifying their pregnancy monitoring during the first COVID-19-related lockdown in France, our study also aimed to stress the declared reasons for these voluntary postponements or waiving of care. In addition to those related to the pandemic (i.e., fear of being infected by SARS-CoV-2, compliance with restrictions on movement), some of these self-perceived reasons were organisational in nature, whether related to healthcare provision, or personal organisation problems linked to the pandemic. In Covimater, 28.6, 17.7 and 15.3% of women who voluntarily changed their pregnancy monitoring declared, respectively, that they had not managed to make an appointment, that they had not been able to contact the health professional who usually followed them, or that it had been impossible for them to take days off work to attend their pregnancy appointments during lockdown. Despite French authorities’ recommendations to promote video and telephone-based consultations when possible outside of the three compulsory ultrasounds requiring physical presence, a relatively large proportion of women who modified their pregnancy monitoring did it because they were unable to contact healthcare structures. In terms of personal organisation of healthcare schedules, discussions are currently underway at the national level to provided current and future parents with greater flexibility to better reconcile their professional and parenthood [41].

### Strengths and limitations

To the best of our knowledge, Covimater was the first national study in France to explore the experiences and behaviours of pregnant women during the SARS-CoV-2 pandemic. It used the same methodology as another study conducted in France at the same time on the general population entitled CoviPrev. This choice was made to ensure comparison with women of childbearing age. Unlike studies from other countries which mostly focused on the third trimester of pregnancy during the current pandemic, Covimater included women with different gestational ages. In Covimater, although some groups compared were unbalanced in size (with consequently reduced power), this did not prevent the identification of significant associations with the variable of interest.

Covimater had some limitations. First, the use of a panel and quota sampling could imply a bias in the pregnant women included for the survey. However, there was no alternative method available that would allow for the study to take place within a short time after the lockdown and thus avoid a significant recall bias. The further away the lockdown was, the more difficult it would have been to collect reliable information from women about their behaviour and feelings during the period.. Consequently, greater caution is required when interpreting the statistical inference than would be needed for random sample studies. Second, sampling bias could explain the overestimation of the percentage of pregnant women with pre-existing chronic diseases or obesity. Third, as the study questionnaire was self-administered, there is always the risk that respondents misunderstood or misinterpreted questions and a risk of recall biases or potential social desirability. Thus, the declarative nature of our survey may have led women to indicate changes in their pregnancy monitoring that were in fact a consequence of underlying situations (social desirability bias, self-complacency biais, memory bias, etc.), which could not be assessed. However, there is no reason to suppose that either of these biases should be limited to the particular sub-group of pregnant women who had postponed/forgone their pregnancy monitoring.

## Conclusions

The results of this study highlighted the importance of defining strategies to prevent voluntary changes in pregnancy monitoring among women in difficult social situations (isolation, violence), which may be accentuated by the ongoing COVID-19 pandemic. In addition, it is necessary to increase access to teleconsultations and provider-patient communication channels during pandemics. Despite the continued difficulties that countries around the world are still facing, this period provides an unprecedented opportunity for maternity units and hospitals to rethink their organisations and reinforce access to care for future health crises and ensure effective and efficient follow-up of pregnant women. One possible strategy - currently being discussed at the national level - is to coordinate all concerned professionals around a reference person [41].

## Supplementary Information

**Additional file 1.** “Covimater - SARS-CoV-2 pandemic, first lockdown and pregnant women – Questionnaire”. Objects offered to pregnant women during the first confinement in France in order to assess, among other things, their pregnancy monitoring.

## Data Availability

The datasets generated and/or analysed during the current study are not publicly available due privacy or ethical restrictions but are available from the corresponding author on reasonable request.

## Abbreviations

SARS-CoV-2: Severe acute respiratory syndrome coronavirus 2
COVID-19: Disease caused by SARS-CoV-2
MERS-CoV: Middle East respiratory syndrome-related coronavirus
SPC: Socio-professional category
PR: Prevalence Ratio
NPS: National Perinatal Survey
