# Supplementary material for: Impact of the SARS-CoV-2 pandemic and first lockdown on pregnancy monitoring in France: the COVIMATER cross-sectional study
Source: BMC Pregnancy Childbirth. 2021 Nov 30;21:799. doi: 10.1186/s12884-021-04256-9 (PMC8630988; doi:10.1186/s12884-021-04256-9)
Supplement: Supplementary file 1 — Additional file 1. “Covimater - SARS-CoV-2 pandemic, first lockdown and pregnant women – Questionnaire”. Objects offered to pregnant women during the first confinement in France in order to assess, among other things, their pregnancy monitoring. [file 12884_2021_4256_MOESM1_ESM.pdf]

Source : Santé Publique France

COVIMATER - SARS-CoV-2 pandemic, first lockdown and pregnant women Questionnaire

Elements related to programming:

- Mandatory answer to each question
- 1 answer per question unless specifically instructed
- No way back

Quotas and markings

To whole panel

Z1. Were you pregnant during the lockdown or part of the lockdown (i.e. between March 17 and May 11)?

1. Yes
2. No ➔STOP QUESTIONNAIRE

To all pregnant women

Z2. When did you give birth or when are you due to give birth?

If you don't know, please indicate an approximate date

/__/__/ /__/__/ /__2/_0_//__/__/

Day Month Year

➔ Stop questionnaire if:

Date of delivery before 01/04/2020

Theoretical delivery date after 01/01/2021

If Z2 <or= today’s date then RecodZ2 = 1

If Z2 > today's date then RecodZ2 = 2

RecodZ2 Status of women

1. Delivered
2. Not yet given birth

To women who have delivered

Z2b. Did you give birth to a live-born child?

1. Yes
2. No

➔ If the response is “no”, do not present the questions about the child postnatally and add the text "The following questions are about the course of your pregnancy, so you will be able to answer them, but you can also decide to stop now if you prefer"

Women who have delivered

Z3a. Did you give birth to a premature baby?

1. Yes
2. No

If women delivered a premature baby
Z3b. At how many weeks of amenorrhea or at what month of pregnancy did you give birth?
Range: 23 - 37
/__/__/ weeks of amenorrhea
Or
Range: 5 - 8.5
/__/__/./__/ months

☐ I don't know

If not yet given birth
Z4a How many children do you have, including your partner's children if they live with you frequently (not including the one you are expecting)?
If you do not have any, indicate 0

If delivered
Z4a How many children do you have, including your partner's children if they live with you frequently (including the newborn child)?

Range: 0-20 (0 only possible if not yet given birth)
/__/__/ child(ren)

If at least one child (Z4a>0)
If she has not yet given birth and has no children then do not ask the question and automatically recode the age of the child in 0
Z4b Precisely how old are your children?
If your child is less than 1 year old, indicate 0
Range: 0-40
a) 1st child: Age: /__/__/ years
b) 2nd child: Age: /__/__/ years
c) 3rd child: Age: /__/__/ years
d) …

Women with multiple children who have not yet given birth
Z4c. Is the upcoming delivery your first one?
Women with multiple children who have delivered
Z4c. Was this your first delivery?
1. Yes
2. No

Deduce the answers to the variable below according to those already given above (childbirth's status, having already had one or more children, having already given birth).
Recoding of primiparous or multiparous parents
(quotas)
1. Primipare
2. Multipare

To all
Z5. How old are you now?
Range: 18-55

/__/__/ years

To all
Z6a Please indicate the postal code of your usual place of residence

/__/__/__/__/__/
If postal code does not exist, display blocking error message

If postal code corresponds to several municipalities
Z6b. In which town do you live?
List of municipalities according to postal code
NB : Z6a and Z6b were not transmitted to Santé publique France.

Recoding from Z6a
Recod REGION ZEAT in 12
(quotas)
1. Ile-de-France
2. Centre-Val-de-Loire
3. Bourgogne-Franche-Comte
4. Normandy
5. Nord-Pas-de-Calais-Picardie (Hauts de France)
6. Alsace-Champagne-Ardenne-Lorraine (Grand-Est)
7. Pays-de-la-Loire
8. Bretagne
9. Aquitaine-Limousin-Poitou-Charentes (Nouvelle Aquitaine)
10. Languedoc-Roussillon-Midi-Pyrénées (Occitanie)
11. Auvergne-Rhône-Alpes
12. Provence-Alpes-Côte-Azur and Corse

Recoding from Z6b
Recod AGGLOMERATION SIZE
(quotas)
1. Rural
2. 2 000 à 20 000
3. 20 000 à 99 000
4. 100 000 and more
5. Paris area

To all
Z7. What is your current situation (or what was it before your maternity leave)?
1. Employee
2. Self-employed
3. Looking for a first job (you have never worked)
4. Looking for a job (you have already worked)
5. At home
6. Student
7. Another situation

Z7b. What is your profession/socio-professional category (or was what it before your maternity leave)?

Z7b. Before you became unemployed, what was your last occupation/socio-professional category?
1. Farmer
2. Craftswoman, Tradeswoman
3. Head of a company with more than 10 employees
4. Liberal profession (EXCEPT paramedical)
5. Teacher / scientific profession
6. Executive or other senior professional
7. Foreman, supervisor, paramedic, technician
8. Schoolteacher
9. Employee
10. Service personnel
11. Labourer
12. Student
13. Other inactive

If Z7b=1or2or3 then recodZ7=1
If Z7b=4or5or6 then recodZ7=2
If Z7b=7or8 then recodZ7=3
If Z7b=9or10 then recodZ7=4
If Z7b=11 then recodZ7=5
If Z7b=12 then recodZ7=6
If Z7b=13 then recodZ7=7
recodZ7 : PROFESSION INTERVIEWED IN 7 POSITIONS
1. self-employed business owners
2. executives and senior professional
3. middle occupations
4. employees
5. female labourer
6. pupils/students
7. other miscellaneous inactive

If Z7b=1or2or3or4or5or6or7or8 then recodZ7b=1
If Z7b=9or10or11 then recodZ7b= 2
If Z7b=12or13 then recodZ7b = 3
recodZ7b : PROFESSION INTERVIEWED IN 3 POSITIONS
(quotas)
1. CSP +
2. CSP -
3. Inactive

To women with Z7 = 1or2or3or4or6
DIV_1. DURING LOCKDOWN, most often during the week...
If your situation varied during the lockdown, please indicate which one lasted the longest
1. You worked from home
2. You worked at your usual place of work (not at home)
3. You worked from home and at your usual place of work (excluding at home)
4. You did not work because you were unemployed (COVID-related or not)
5. You did not work for other reasons (maternity leave, sick leave, etc.)
6. Other situation

If DIV_1= 1or2or3
DIV_2. DURING LOCKDOWN, how was your workload?

1 - It was lighter than usual
2 - It was heavier than usual
3 - It was the same as usual

The following questions are about the people you were with and where you lived during the lockdown. If your situation varied during the lockdown, please indicate which one lasted the longest.

To all
Z8. DURING LOCKDOWN, who did you live with?
Several answers possible except “alone”

1. With your spouse
2. With your child(ren) (To women who did not answer 0 in Z4a)
3. With one or both of your parents
4. With other members of your family (uncle, aunt, grandparent, cousin, parent-in-law, nephew...)
5. With friends or acquaintances
6. Alone

To all except women living alone (Z8=6)
Z9a. DURING LOCKDOWN, were you with the people you usually live with?

1. Yes
2. No

To all
REL_1. DURING LOCKDOWN, would you say that you were...

1. Very well supported
2. Well supported
3. Little supported
4. Not supported at all

To all
REL_2. DURING LOCKDOWN, did you experience any serious disputes or violence?

1. Very often
2. Often
3. Sometimes
4. Rarely
5. Never

To all
Z9b. DURING LOCKDOWN, were you in your usual place of residence?

1. Yes
2. No
3.

To all
Z10a. DURING LOCKDOWN, were you living in...

1. A house
2. An apartment in a shared building/block of flats
3. Other: emergency accommodation, care home, hotel, caravan, ...

To all

Z10b. DURING LOCKDOWN, did you have an outdoor space in your place of residence, whether private or shared (garden, balcony, terrace, etc.)?

1. Yes
2. No

General information about your pregnancy

For women who have not given birth
GRO_1a. You are expecting...
For women who have given birth
GRO_1b: You gave birth to...

1. one baby
2. twins or triplets

To all
GRO_2 BEFORE YOU WERE PREGNANT, did you have any chronic or long-term illnesses?

Multiple choices possible except for "No health problems or chronic illness"
Random rotation of items except "No health problems or chronic illness"

1. Diabetes
2. Hypertension
3. Heart disease
4. Asthma
5. Obesity
6. Overweight
7. Coagulation disorder
8. Autoimmune disease (multiple sclerosis, hypo/hyperthyroidism...)
9. A mental illness (manic/bipolar disorder...)
10. Other, please specify______________________
11. No health problems or chronic illnesses

To all
GRO_3 DURING THIS PREGNANCY, were you told that you had an illness related to your pregnancy?

Multiple choices except No pregnancy-related illness
Random rotation of items except No pregnancy-related illness

1. Gestational diabetes
2. Hypertension in pregnancy
3. A threat of premature delivery
4. Other, please specify___________________________
5. No pregnancy-related pathology

To all
GRO_4. DURING LOCKDOWN, did you personally feel that you were at high risk from Coronavirus (COVID-19)?
Give a score between 0 and 10: 0 indicates that you did not think you were clinically vulnerable at all and 10 indicates that you thought you were highly vulnerable.
The intermediate scores allow you to grade your answer.

0		1	2	3	4	5	6	7	8	9	10

I don't know

Your perception of the Coronavirus (COVID-19) pandemic

To all
PERC_1. DURING LOCKDOWN, were you generally worried about the Coronavirus (COVID-19) pandemic in France?
Give a score between 0 and 10: 0 means you were not at all worried and 10 means you were very worried.
The intermediate scores allow you to grade your answer.

0	1	2	3	4	5	6	7	8	9	10

I don't know

To all
PERC_2. DURING LOCKDOWN, were you especially worried about...
Random rotation of items - One answer per item

a) Catching COVID-19 and having severe symptoms
b) Transmitting the Coronavirus to your baby and the consequences for him/her
c) The people you were in lockdown with being contaminated (Only to women who did not live alone)
d) Vulnerable loved ones catching the Coronavirus (elderly or previously sick)
e) Having to change your antenatal care
f) Going to the maternity hospital for antenatal care, check-ups or to give birth
g) Not being able to benefit from childbirth preparation sessions
h) Not being able to prepare for your baby's arrival at home as you would have liked
i) Having to give birth alone without your spouse or a support person
j) Having to give birth in unusual conditions
k) The impact of the pandemic on the stay in the maternity
   1) Yes, absolutely
   2) Yes, slightly
   3) No, not really
   4) No, not at all

Pregnancy monitoring

We will now discuss the antenatal care during your pregnancy

Women whose pregnancy started before lockdown
FOLLOW-UP_1. JUST BEFORE LOCKDOWN, which health professional(s) mainly followed your pregnancy?

If pregnancy started during lockdown
FOLLOW-UP_1. DURING LOCKDOWN, which health professional(s) mainly followed your pregnancy?

Several answers possible except 9

1. A gynecologist or obstetrician in an urban practice
2. A gynecologist or obstetrician in a public or private maternity hospital
3. A GP
4. A midwife in town
5. A midwife in a public or private maternity hospital
6. A midwife in a local maternity clinic
7. A doctor in a local maternity clinic
8. A doctor or a midwife at the PMI (mother and child protection centre)
9. I hadn't started my antenatal care

All except women with early pregnancy during lockdown
When FOLLOW-UP_1=9 (automatically recode FOLLOW-UP_2=2)
FOLLOW-UP_2. DURING LOCKDOWN, did you have any scheduled appointments to monitor your pregnancy?

1. Yes
2. No

To all
FOLLOW-UP_3. DURING LOCKDOWN, did you...

a) Have consultations postponed or cancelled by the hospital or the professionals who followed you before the lockdown
b) Voluntarily cancel any consultations
c) Voluntarily postpone any consultations
d) Benefit from teleconsultations (video or telephone) for pregnancy monitoring
e) Benefit from childbirth preparation sessions by video or telephone
f) Receive antenatal care from a health professional other than the one who usually follows you (do not ask women with FOLLOW-UP_1=9)
g) Change your birth plan (whether written or unwritten) due to the pandemic
    1. Yes
    2. No

All except women who hadn't started their pregnancy monitoring and women with early pregnancy during lockdown

FOLLOW-UP_4. Because of the lockdown, did you forgo...

a) Gestational Diabetes screening test
b) Streptococcus B screening test
c) Trisomy 21 (Down syndrome) screening test
d) Mandatory blood screenings for the declaration of pregnancy (rubeolla, toxoplasmosis, syphilis)
e) Hepatitis B screening
f) The 1st pregnancy ultrasound (scheduled between the 11th and 13th week of pregnancy)
g) The 2nd pregnancy ultrasound (scheduled between the 22nd and 24th week of pregnancy)
h) The 3rd pregnancy ultrasound (scheduled between the 31st and 33rd week of pregnancy)
i) One or more additional ultrasounds in the context of a risky pregnancy monitoring (pathology, growth monitoring...)
j) Monthly follow-ups for toxoplasmosis serology if you were not immune
k) The declaration of your pregnancy within the legal time frame (during the first three months of pregnancy in France)
l) Other additional exams/consultations that were prescribed or recommended to you

1. Yes, I turned it down
2. No, I did it
3. No, because it was not offered to me during lockdown
4. I was not concerned

If at least one Yes item in FOLLOW-UP_3 a, b, c, f, g or at least one Yes in FOLLOW-UP_4

FOLLOW-UP_5. DURING LOCKDOWN, you changed your antenatal care because....

Multiple responses possible Random rotation of items

a) You were worried about catching the Coronavirus (COVID-19)
b) You had to take care of your other children (only if Z4>0 if has not yet given birth RecodZ2=2 or if Z4>1 if has delivered or RecodZ2=1))
c) You were confined away from your usual residence (only if Z9b=2)
d) It was recommended not to leave home
e) You didn't want to use public transport
f) You didn't want to go to a doctor's surgery
g) You did not want to go to a hospital or clinic
h) You were not able to reach the health professional in charge of your antenatal care
i) You couldn’t make an appointment
j) You didn't know what measures were available to protect you during consultations
k) You didn't have a mask
l) You couldn’t participate in teleconsultations
m) It was impossible for you to take time off work or to be absent from your workstation to attend your pregnancy monitoring (Only for women who worked during the lockdown DIV_1=1or2or3)
    1. Yes
    2. No

To all

FOLLOW-UP_6. DURING LOCKDOWN, did you talk to a health professional about...

a) The risk of becoming infected with the Coronavirus and having severe symptoms
b) The risk of transmitting the Coronavirus to your baby and the consequences for him

c) Pregnancy monitoring during the pandemic
d) The delivery course during the pandemic
e) The course of maternity stay during the pandemic
f) The ability to breastfeed safely during the pandemic
    1. Yes
    2. No because I didn't need it
    3. No, but I would have liked to

To all
FOLLOW-UP_7. DURING LOCKDOWN, due to restrictions related to the Coronavirus (COVID-19) pandemic, has was a situation where YOUR SPOUSE or a SUPPORT PERSON could not join you for...

One answer per item
a) A pregnancy ultrasound
b) A consultation monitoring at the hospital or clinic
c) A consultation monitoring in an urban practice
d) A childbirth preparation session
e) A biological examination (blood test...)
f) (To women who have delivered) Your delivery

    1. Yes, it happened to me
    2. No, this never happened to me
    3. Not concerned, I did not wish to be accompanied

To all
FOLLOW-UP_8. Overall, would you say that the lockdown had an impact on...
a) (To all) your antenatal care
b) (To women who have given birth) how your delivery went
c) (To women who have given and a child born alive) the medical monitoring of your newborn
d) (To all) Your wish to breastfeed or not
    1. Yes, a very significant impact
    2. Yes, quite a significant impact
    3. Yes, but low impact
    4. No, no impact

Lockdown situation

We will now talk about your experience of lockdown.

To all
VEC_1. Overall, do you feel that lockdown has been...

1. An unusual period but nothing remarkable
2. A really trying time
3. A period with pleasant but also difficult moments
4. An opportunity (to take your time, to prepare for the arrival of the baby, to enjoy your loved ones...)

To all
VEC_2. JUST BEFORE the lockdown, psychologically, how did you feel?

1 - Good
2 - Quite good
3 - Quite poor
4 - Poor

To all
VEC_3. DURING LOCKDOWN, psychologically speaking, how did you feel?

1 - Good
2 - Quite good
3 - Quite poor
4 - Poor

At all
VEC_4. DURING LOCKDOWN, did you feel the following emotions more strongly than usual?
a) Relief
b) Fatigue
c) Frustration
d) Serenity
e) Anger
f) Security
g) Powerlessness
h) Despair
i) Loneliness
j) Fear

1. Yes, a lot
2. Yes, somewhat
3. No, not really
4. No, not at all

To all
VEC_5. BEFORE lockdown, had you been followed by a psychiatrist, a psychologist or another professional (general practitioner, psychotherapist...) for more than 6 months for a psychological problem?

1. Yes
2. No

To all
VEC_6. DURING LOCKDOWN, did you call on a psychiatrist, psychologist or other professional (general practitioner, psychotherapist...) to provide you with the psychological support you needed?

1. Yes
2. No because I did not need it
3. No but I would have liked to

To all
VEC_7. BEFORE lockdown, had you ever taken, for a period of 6 months or more, medication for a mood problem (depression, anxiety...) or a sleep disorder, such as tranquillizers, sleeping pills or antidepressants?

1. Yes
2. No

To all
VEC_8. DURING LOCKDOWN, did you take medication for a mood problem (depression, anxiety...), or a sleep disorder, such as tranquillizers, sleeping pills or antidepressants?

1. Yes
2. No because I did not need it
3. No but I would have liked to

Nutrition - Sleep - Sports activity

We will now talk about your diet and your consumption of tobacco and alcohol during lockdown.

To all
Ali1. BECAUSE OF LOCKDOWN...
a) In terms of quantity, you ate
b) You had a balanced diet
c) You followed the rules of hygiene to prevent toxoplasmosis and listeria (avoid raw meat, raw milk, cold meats, wash vegetables soiled by soil...)
d) You paid attention to your weight gain
e) You ate fruit and vegetables
f) You ate fatty, salty or sweet products
g) You drank tea or coffee
h) You had problems sleeping
i) You practiced sports activities adapted to your condition as a pregnant woman

1. More than before the lockdown
2. Less than before the lockdown
3. As before the lockdown, I didn’t change anything

At all
Ali2. How tall are you?
Specify your height in inchs - Example: 59 inches
Please answer approximately if you don't know
Boundaries: 39 -78

l__l__l__l inches

At all
Ali5. What was your weight before pregnancy?
Specify your weight in kilos (kg)
Please answer approximately if you do not know
Range: 30-170

/__/__/__//__/ kg

To women who have not given birth
Ali7. What is your current weight?
To women who have given birth
Ali7. What was your weight at the time of delivery?
Specify your weight in kilos (kg)
Please answer approximately if you do not know
Range: 30-170

/__/__/__//__/ kg

Tobacco consumption

At all

Tab1. Just before lockdown, did you smoke?

1. Yes, cigarettes (apart from electronic cigarettes), including rolled cigarettes
2. Yes, only other types of tobacco (chicha...)
3. No, I had stopped smoking because I was pregnant or in anticipation of pregnancy
4. No, I had not smoked for a long time before becoming pregnant or planning to become pregnant
5. No, I did not smoke (or very occasionally)

If smoked cigarettes before lockdown
Tab2. Just before lockdown, on average, how many cigarettes did you smoke? (including rolled cigarettes)
Please indicate even an approximate number of cigarettes and do not include electronic cigarettes
Answer in 1 or 2 or 3 or 4 - Boxes: 1 to 100

1. /__/__/__/ per day
2. /__/__/__/ per week
3. /__/__/__/ per month

To smokers before lockdown
Tab3a DURING LOCKDOWN, how did your tobacco consumption (cigarettes, rolled cigarettes, apart from electronic cigarettes) change?

1. It increased
2. It remained stable
3. It decreased
4. I stopped smoking

To non-smokers before lockdown
Tab3b DURING LOCKDOWN, did you start smoking or start again after quitting cigarettes (including rolled cigarettes and excluding electronic cigarettes)?

1. Yes
2. No

If smoked during lockdown
Tab4. DURING LOCKDOWN, on average, how many cigarettes did you smoke? (including rolled cigarettes)
Please indicate even an approximate number of cigarettes and do not include electronic cigarettes
Answer in 1 or 2 or 3 - Terminals: 1 to 100

1. /__/__/__/ per day
2. /__/__/__/ per week
3. /__/__/__/ per month

If smoker increased her smoking during lockdown
Tab5. DURING LOCKDOWN, for what reason(s) did you increase, start or resume smoking cigarettes (including rolled cigarettes)?

Source : Santé Publique France

Several answers possible

1. Stress
2. Boredom, lack of activity
3. For pleasure
4. I was following a substitution treatment (patches, gums...) which I had to stop
5. I was using an electronic cigarette and I ran out of liquid to refill it or it broke down
6. I no longer had any psychological support
7. Proximity to other smokers
8. For another reason

If smoker having reduced her tobacco consumption during lockdown
Tab6. DURING LOCKDOWN, for what reason(s) did your smoking decrease?
If ex-smoker since lockdown
DURING LOCKDOWN, for what reason(s) did you stop smoking cigarettes (including rolled cigarettes)?
Multiple answers possible

1. For the health of the people who live with me (children, spouse, etc.)
2. For my health
3. For the health of my baby
4. The lockdown was a good time to smoke less or stop smoking
5. I was already trying to cut down or quit smoking before the lockdown
6. I knew I was pregnant during the lockdown
7. I was less stressed than usual
8. I had difficulty getting cigarettes or tobacco
9. I had fewer opportunities to smoke with friends or colleagues (fewer social or festive occasions)
10. I started using electronic cigarettes
11. For another reason

Alcohol consumption

To all
Alc1. DURING LOCKDOWN, how did your consumption of alcoholic beverages (beer, wine, cider, hard liquor, champagne or any other type of alcohol, even low alcohol content) change?

1. It increased compared to before the lockdown
2. It remained stable compared to before the lockdown
3. It decreased compared to before the lockdown
4. I never drink alcohol

If alc1=1,2,3
Alc2. DURING LOCKDOWN, how often did you drink alcoholic beverages (beer, wine, cider, hard liquor, champagne or any other type of alcohol, even low alcohol content)?

1. Never
2. 1 time a month or less
3. 2 to 4 times a month
4. 2 to 3 times a week
5. 4 or more times a week, but not every day
6. Every day

If Alc2 different from 1
Alc3. DURING LOCKDOWN, how many glasses of alcoholic beverages did you drink in a week (including weekends)?

1. Less than 1 drink
2. 1 to 4 drinks
3. 5 to 10 drinks
4. 11 to 13 drinks
5. 14 or more drinks

Knowledge of symptoms and modes of transmission

We will now ask you about the Coronavirus and how you protected yourself from it

To all
QCONN_1. Here are several statements about the Coronavirus (COVID-19). Please indicate for each one whether you think it is true or false.
One answer per item
a) The virus can be transmitted by people who have no symptoms
b) The virus survives on inert surfaces such as door handles or bars in public transport
(c) It is possible to become infected in closed public places such as food shops or on public transport
d) It is possible to be contaminated in open public places such as a park or a beach
(e) There is a greater risk of being contaminated when you are less than 1 metre away from a person
f) There is less risk of being contaminated if everyone wears a mask
g) There is less risk of being contaminated if everyone washes their hands regularly

1. True
2. False

Protective measures applied

At all
PROT-1. Did you adopt any of the following protective measures...
One answer per item
Ask item d and e only to women who worked during the lockdown

	DURING LOCKDOWN	AT PRESENT
a) Wearing a mask in public	1. Yes, systematically<br>2. Yes, often<br>3. Yes, occasionally<br>4. No, never	1. Yes, systematically<br>2. Yes, often<br>3. Yes, occasionally<br>4. No, never
b) Avoiding public transport	1. Yes, systematically<br>2. Yes, often<br>3. Yes, occasionally<br>4. No, never	1. Yes, systematically<br>2. Yes, often<br>3. Yes, occasionally<br>4. No, never
c) Staying at home as much as possible	1. Yes, systematically<br>2. Yes, often<br>3. Yes, occasionally<br>4. No, never	1. Yes, systematically<br>2. Yes, often<br>3. Yes, occasionally<br>4. No, never
d) Choosing to work at home rather than at work	1. Yes, systematically<br>2. Yes, often<br>3. Yes, occasionally<br>4. No, never	1. Yes, systematically<br>2. Yes, often<br>3. Yes, occasionally<br>4. No, never
e) Adapting your workstation to limit the risk of infection	1. Yes, systematically<br>2. Yes, often<br>3. Yes, occasionally<br>4. No, never	1. Yes, systematically<br>2. Yes, often<br>3. Yes, occasionally<br>4. No, never
f) Washing your hands very regularly with soap or using hydroalcoholic gel	1. Yes, systematically<br>2. Yes, often<br>3. Yes, occasionally<br>4. No, never	1. Yes, systematically<br>2. Yes, often<br>3. Yes, occasionally<br>4. No, never
g) Avoiding gatherings and face-to-face meetings with relatives who do not live with me (family reunions or meetings with friends, aperitifs, discussions between neighbours, etc.)	1. Yes, systematically<br>2. Yes, often<br>3. Yes, occasionally<br>4. No, never	1. Yes, systematically<br>2. Yes, often<br>3. Yes, occasionally<br>4. No, never
h) Keeping a distance of at least one metre from other people in shops, markets, public places or outdoors	1. Yes, systematically<br>2. Yes, often<br>3. Yes, occasionally<br>4. No, never	1. Yes, systematically<br>2. Yes, often<br>3. Yes, occasionally<br>4. No, never

COVID-19 experience

At all
EXP_1. Have you yourself had any symptoms or signs of illness since January 2020 that made you think of the Coronavirus (COVID-19)?

1. 1. Yes
2. 2. No

If had symptoms of coronavirus
EXP_2. When did these symptoms or signs of the disease start?

1. Before lockdown (before 17 March)
2. During lockdown (from 17 March to 11 May)
3. After lockdown (after 11 May)

If had symptoms of coronavirus
EXP_3. Following these symptoms or signs of the disease, what did you do?
Multiple answers possible

1. I called a doctor
2. I went to a doctor for a consultation
3. I called 15, the emergency services number
4. I went to the general A&E ward
5. I went to the maternity emergency ward
6. I was hospitalised
7. I didn't do any of these things

At all
EXP_4. Were you tested to see if you had the Coronavirus (blood test / nose, mouth or throat swab)?

1. Yes and it confirmed the Coronavirus
2. Yes and it was not the Coronavirus
3. Yes, but I haven't received the results yet
4. No

At all
EXP_5. Have any of your friends or family had the Coronavirus or any signs of illness suggesting that it was the Coronavirus?

1. Yes
2. No

Mental health: Anxiety (A) and depressive (D) disorders
The following questions are about your state of mind at the moment

At all
Q330_A. Choose the answer that best describes how you have felt over the past few days:
I feel tense or 'wound up':
1. Most of the time
2. A lot of the time
3. From time to time, occasionally
4. Not at all

At all
Q330_D. In the past few days, I still enjoy the things I used to enjoy
1. Definitely as much
2. Not quite so much
3. Only a little
4. Hardly at all

At all
Q331_A. In the past few days, I get a sort of frightened feeling as
if something awful is about to happen:
1. Very definitely and quite badly
2. Yes, but not too badly
3. A little, but it doesn't worry me
4. Not at all

At all
Q331_D. In the past few days, I can laugh and see the funny side of things:
1. As much as I always could
2. Not quite so much now
3. Definitely not so much now
4. Not at all

At all
Q332_A. In the past few days, worrying thoughts go through my mind:
1. A great deal of the time
2. A lot of the time
3. From time to time but not too often
4. Only occasionally

At all
Q332_D. In the past few days, I feel cheerful
1. Not at all
2. Not often
3. Sometimes
4. Most of the time

At all
Q333_A. In the past few days, I can sit at ease and feel relaxed:
1. Definitely

2. Usually
3. Not often
4. Not at all

At all
Q333_D. In the past few days, I feel as if I am slowed down:
1. Nearly all the time
2. Very often
3. Sometimes
4. Not at all

At all
Q334_A. In the past few days, I get a sort of frightened feeling like 'butterflies' in the stomach:
1. Not at all
2. Occasionally
3. Quite often
4. Very often

At all
Q334_D. In the past few days, I have lost interest in my appearance:
1. Definitely
2. I don't take so much care as I should
3. I may not take quite as much care
4. I take just as much care as ever

At all
Q335_A. In the past few days, I feel restless as if I have to be on the move…
1. Very much in deed
2. Quite a lot
3. Not very much
4. Not at all

At all
Q335_D. In the past few days, I look forward with enjoyment to things :
1. As much as ever I did
2. Rather less than I used to
3. Definitely less than I used to
4. Hardly at all

At all
Q336_A. In the past few days, I get sudden feelings of panic
1. Very often indeed
2. Quite often
3. Not very often
4. Not at all

At all
Q336_D. In the past few days, I can enjoy a good book or radio or TV programme
1. Often

2. Sometimes
3. Not often
4. Very seldom

Descriptive information (end of questionnaire)

At all

Z11. Finally, here are some last questions about you or your household.

What was the last qualification you obtained?

1. I have no qualifications
2. Certificate of primary education
3. BEPC - Brevet des collèges [secondary school leaver]
4. CAP / BEP [vocational diploma]
5. BAC / Brevet professionnel / Brevet de technician [A-levels/BTEC]
6. BAC +2 and more [university degree]

At all

Z12. At present, would you say that financially...

1. You are comfortable
2. You are managing
3. It's tight, you have to be careful
4. It’s hard to get by
5. You can't get by without going into debt

At all

Z13.a Currently, do you have ....

a. Social security coverage for health (national insurance)
b. Supplementary health insurance
    1. Yes
    2. No

At all

Z13.b Currently, do you benefit from...

Several answers possible

1. The Complementary Health Insurance (CSS) (previously called CMUc or ACS)
2. State Medical Aid (AME)
3. Revenu Sociale d'Activité (RSA) [universal revenue]
4. Jobseekers allowance (ARE)
5. No

At all

Z14. Were you born in France (metropolitan France, overseas departments or regions)?

1. Yes
2. No

To women who answered “No” in Z14

Z15. In which country were you born?

/___________________/
